# Fixation of an Osteochondral Fracture of the Lateral Femoral Condyle With Knotless Suture Anchors

**DOI:** 10.1016/j.eats.2025.103715

**Published:** 2025-06-30

**Authors:** Julian M. Moore, Daniel W. Hogan, Clayton W. Nuelle, Steven F. DeFroda

**Affiliations:** a Arizona College of Osteopathic Medicine, Midwestern University, Glendale, Arizona, U.S.A.; b Department of Orthopaedic Surgery, University of Missouri, Columbia, Missouri, U.S.A.

## Abstract

Osteochondral fractures of the knee involve disruption of the subchondral bone and overlying cartilage, which can lead to instability and early osteoarthritis. These lesions often require immediate surgical fixation to preserve joint surface anatomy. Although several fixation methods exist, there is no universally accepted gold standard. Thin bony fragments often limit the effectiveness of traditional implants. We describe a knotless suture anchor technique (PushLock; Arthrex, Naples, FL) to achieve stable fixation and restore articular congruity in an unstable, traumatic osteochondral fracture.

Osteochondral fractures (OCFs) of the knee are acute injuries that typically occur in adolescents and young athletes after a torsional pivoting force or direct axial loading of the femoral condyles. The susceptibility of younger patients to present with these fractures is attributed to general ligamentous laxity and decreased biomechanical strength at the osteochondral junction.^1^ Lateral dislocation of the patella is present in most of these fractures.^2^^,^^3^

Traumatic OCFs with displaced fragments and mechanical symptoms require prompt surgical intervention to restore joint congruity and preserve hyaline cartilage.^2^^,^^3^ In contrast to osteochondral dissecans lesions, OCFs are often larger and acutely unstable and involve critical load-bearing surfaces such as the weight-bearing lateral femoral condyles (LFCs). Failure to achieve stable fixation can lead to the development of fibrocartilage at the defect site, which lacks the mechanical durability and load-bearing characteristics of native hyaline cartilage and may accelerate joint degeneration.^4^ As such, achieving fragment preservation and anatomic reduction is critical to optimizing outcomes.

Traditional techniques for internal fixation of larger fractures involve metal or bioabsorbable screws, pins, or osteochondral plugs. Although effective, each method carries limitations including hardware prominence, the need for removal, biological reactivity, and cartilage thinning.^5^, ^6^, ^7^ Bioabsorbable pins have widespread use but can result in breakage, migration, and potential failure when used alone.^8^, ^9^, ^10^

Knotless suture anchor fixation has emerged in the past decade as a solution for osteochondral lesions.^2^^,^^5^ This technique offers several advantages over traditional hardware-based methods by eliminating protruding implants that can compromise articular cartilage or require later removal. It allows for secure fragment reduction without concentrating stress at a single point, and the low-profile nature of knotless fixation reduces the risk of joint irritation. This approach may be especially beneficial for small osteochondral fragments by eliminating some of the major complications encountered with bioabsorbable pins and metal implants.^8^^,^^9^ Cartilage-preserving, hardware-sparing techniques such as this support early mobilization and have shown promising short-term union rates and functional outcomes.^2^ Long-term joint function improvement may be seen, particularly in adolescent knees and fragments less amenable to screw fixation.

## Surgical Technique

### Patient Positioning

After induction of general anesthesia, the patient is positioned supine on a radiolucent operating table. A lateral post is positioned 1 cm proximal to the patient’s knee. Physical examination under anesthesia is performed per surgeon preference. The limb is exsanguinated, and a thigh tourniquet is inflated to 250 mm Hg.

### Diagnostic Arthroscopy

**Fig 1:**
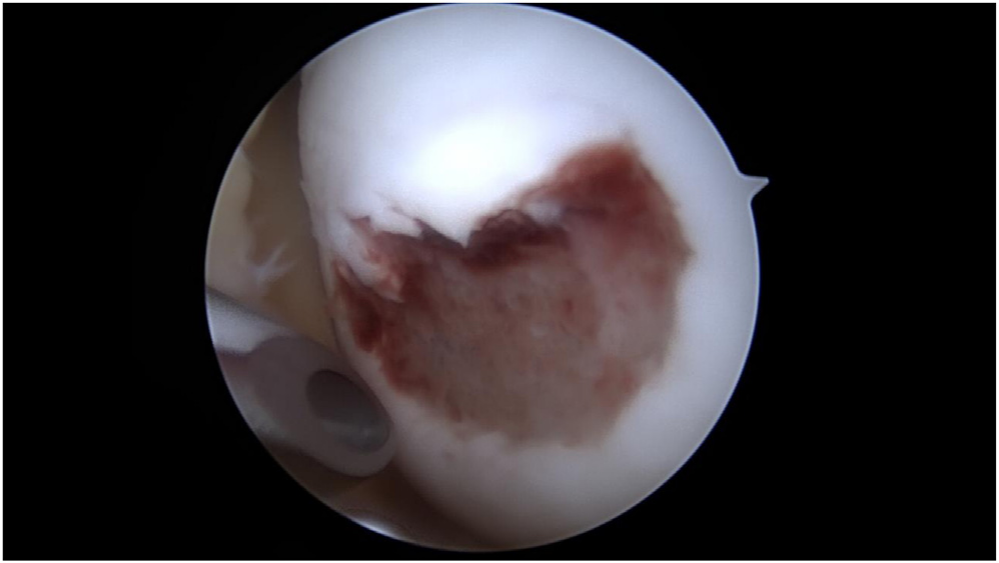
Arthroscopic visualization of a full-thickness lateral femoral condyle defect in a right knee, viewing with a 30° arthroscope from the anteromedial portal. A shaver is introduced to circumferentially debride the osteochondral fracture bed and establish a stable margin of healthy cartilage circumferentially.

**Fig 2:**
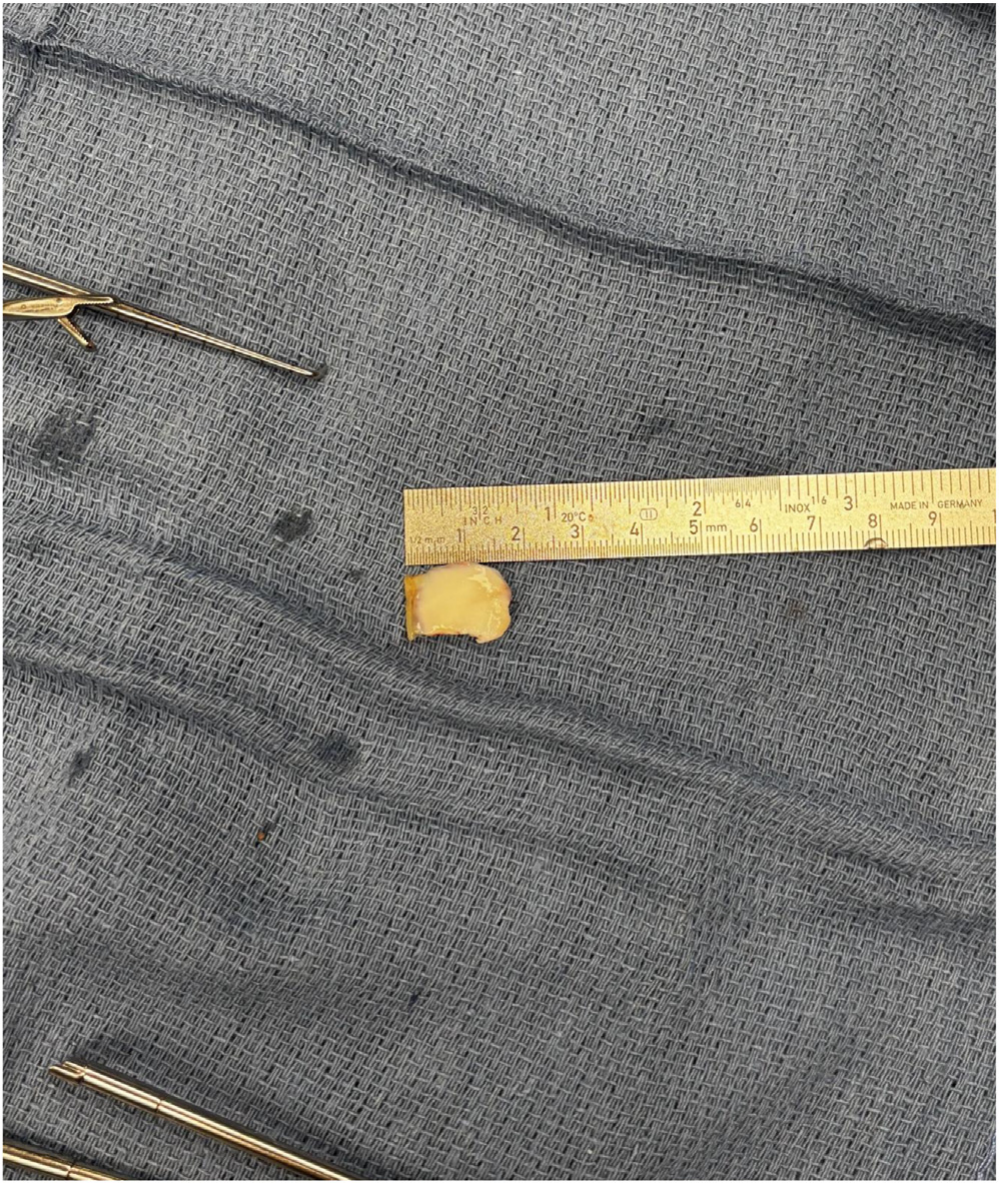
Full-thickness osteochondral fragment of lateral femoral condyle, measuring 1.6 cm × 1.6 cm.

A standard 1-cm anterolateral portal is made, and diagnostic arthroscopy is performed. The cartilaginous defect of the LFC with exposed subchondral bone is identified, and a full-thickness osteochondral fragment is located (Figs 1 and 2). An anteromedial portal is then made under direct visualization. The arthroscope is moved to the anteromedial portal, and a shaver is introduced into the anterolateral portal to debride the subchondral bed and establish a stable margin of healthy cartilage circumferentially. The osteochondral fragment is then removed through the lateral portal as atraumatically as possible and safely preserved on the sterile back table in normal saline solution (Fig 2).

### Open Fixation of Osteochondral Fragment

After diagnostic arthroscopy, a 5-cm midline anterior knee incision is made in standard fashion. Sharp dissection is carried down to the level of the patellar tendon, raising full-thickness flaps medially and laterally. A lateral parapatellar arthrotomy is performed with the knee in hyperflexion. Nonviable cartilage is removed with sharp curettage, resulting in a bleeding bed at the base of the OCF. The subchondral bone at the base of the OCF is then drilled with a 1.6-mm K-wire to promote vascular channels that aid in healing. The chondral fragment can swell with exposure to joint fluid, so it undergoes debulking to fit the anatomic defect on the LFC, and a trial reduction is performed.

Implants are then selected for knotless fixation of the fracture fragment with absorbable suture. After initial predrilling, a 2.5-mm knotless suture anchor (PushLock; Arthrex, Naples, FL) is preloaded with 2 No. 2-0 Vicryl sutures (Ethicon, Somerville, NJ) and positioned at the medial aspect of the LFC defect. The fragment is then reduced and secured in place with a bioabsorbable pin (Trim-It Pin; Arthrex). The pin is cut flush with the chondral surface using a high-temperature disposable cautery pen. Predrilling for the inferolateral 2.5-mm knotless suture anchor is followed by suture fixation with 2 limbs of the previously anchored medial sutures to create a double-row fixation construct. The same steps are performed in a superolateral manner to secure the final 2 limbs of suture, and the sutures are cut flush.

**Fig 3:**
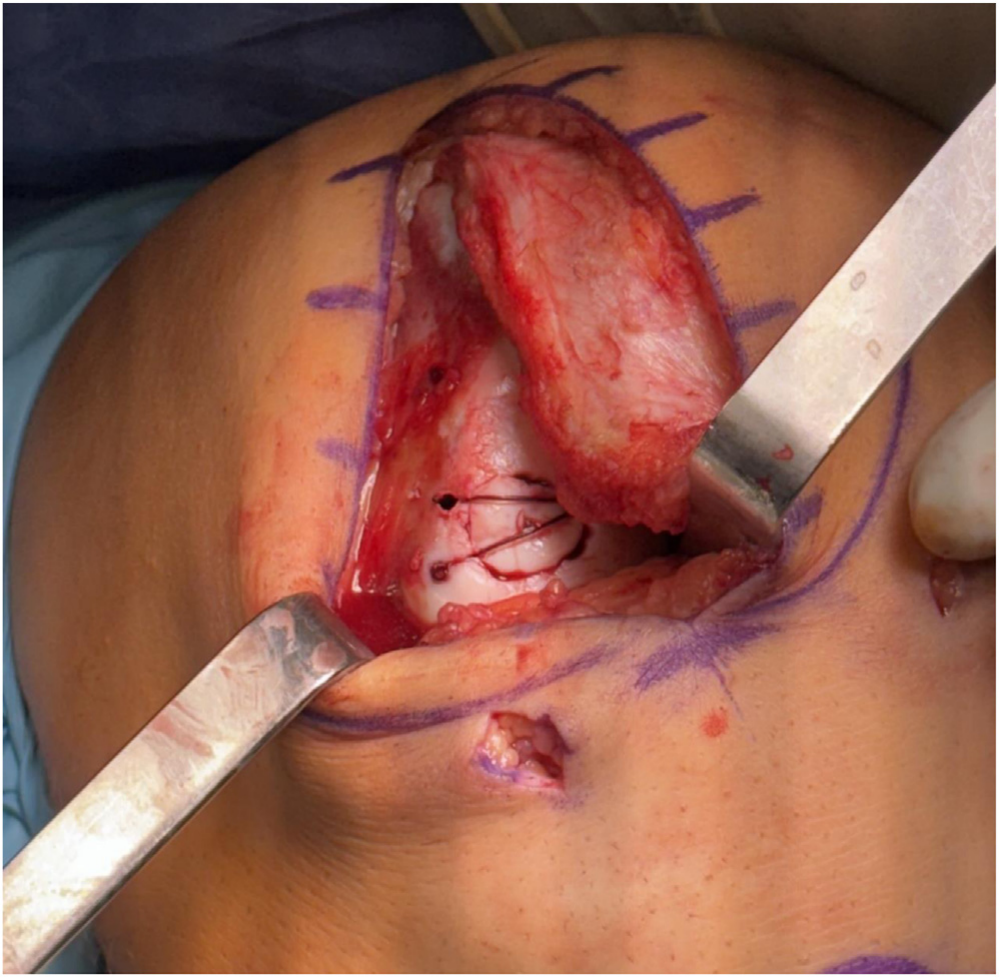
Lateral femoral condyle osteochondral fracture in a right knee viewed through a lateral parapatellar arthrotomy stabilized with knotless suture anchors using absorbable No. 2-0 Vicryl suture.

Range of motion (ROM) is tested to ensure stable fixation and no catching of the chondral fixation. Fibrin glue is then introduced around the perimeter of the defect to promote hemostasis and further secure the final construct (Fig 3).

Additional soft-tissue stabilization procedures such as medial patellofemoral ligament reconstruction are considered based on surgical indication (e.g., patellar dislocation). The tourniquet is then released, and hemostasis is obtained. The wounds are copiously irrigated with normal saline solution. The deep, dermal, and subcuticular layers are closed sequentially. A sterile, soft dressing is then applied. The full technique is demonstrated in Video 1.

### Postoperative Rehabilitation Protocol

Postoperatively, the patient is instructed to bear weight as tolerated with the brace locked at 0° during ambulation and sleeping. Physical rehabilitation begins on postoperative day 1 with a focus on ROM to 30° and lower-limb body weight exercises (i.e., straight leg raise and quadriceps sets). Electrically stimulated muscle activation is also recommended to prevent atrophy and improve postoperative outcomes. ROM gradually increases to 90° by the end of week 6, and the brace is then discontinued.

## Discussion

Our knotless fixation technique for osteochondral fragments of the LFC offers an effective surgical option in cases involving unstable osteochondral fragments with minimal subchondral bone on the fracture fragment. Traditional fixation options, such as bioabsorbable pins or screws, are often limited by prominent hardware in the joint space, the need for later removal, or fragmented lesions.^8^^,^^10^^,^^11^ In contrast, knotless suture anchor fixation enables secure stabilization and minimizes intra-articular bulk, reducing the risk of chondral injury and other hardware-related complications.^2^^,^^12^ There is also no need for future hardware removal. Knotless constructs support early mobilization protocols, which improve joint mechanics and potentially reduce long-term stiffness after fixation.^2^ With the described technique, rehabilitation begins on postoperative day 1 with immediate ROM exercises and muscle activation.

Whereas fragment removal and debridement may be appropriate for small chondral or osteochondral fragments (<5 mm), surgical fixation is recommended for management of larger osteochondral defects.^2^ Similar techniques using knotless suture anchors to obtain firm fixation have been reported.^5^^,^^13^ Knotless suture anchors offer low-profile fixation that reduces the risk of hardware prominence and tissue irritation, often eliminating the need for later hardware removal.^12^ When the osteochondral fragment is salvageable, they provide stable fixation that preserves native cartilage and promotes joint surface restoration.^2^

Our technique uses the PushLock knotless suture anchor in combination with absorbable pins to create a low-profile construct with multipoint triangular fixation. Unlike traditional methods that concentrate force at a single point, this design evenly distributes compression across the osteochondral fragment. Unpublished data cited by Ishibashi et al.^13^ applying this same technique indicate that the initial fixation strength of this construct is greater than that of absorbable pins and osteochondral plugs alone. The technique outlined here may be particularly advantageous for osteochondral lesions of the LFC that are more prone to instability and loose body formation than their medial counterparts.^14^

Nonetheless, this approach has its limitations. Successful outcomes rely on precise anchor placement, appropriate tensioning, and awareness of the bioabsorption profile of all implants.^15^ Advantages and disadvantages of this procedure are highlighted in Table 1, and pearls and pitfalls are presented in Table 2.

**Table 1:** Advantages and Disadvantages of Knotless Fixation of Osteochondral Fracture of Lateral Femoral Condyle

Advantages
Suture can be pulled taut after anchor placement for fine-tuned compression
Learning curve is reduced with less technical demand compared with knot tying
Less intra-articular bulk
Reduction is not limited to a single point of the construct
Disadvantages
Tension must be adequate to avoid displacement of fragment
Pullout strength is potentially less than a knotted construct
The bioabsorbable pin may fracture
Additional costs may vary based on the implant cost and/or number

**Table 2:** Pearls and Pitfalls of Knotless Fixation of Osteochondral Fracture of Lateral Femoral Condyle

Pearls
Precise operative planning of anchor placement maximizes strength of fixation.
Suture loading before lateral anchor insertion maintains fragment control.
The surgeon should confirm that the anchors are flush with the cartilage surface before suture fixation is performed.
Pitfalls
Misaligned anchors can lead to improper anatomic seating of the graft.
Failure to confirm the bioabsorption profile of implants can affect follow-up patient care.
Over-tensioning of the construct should be avoided.

In summary, knotless suture anchor fixation presents a viable alternative to conventional approaches for treating OCFs of the LFC with patellar dislocation. It offers reliable stabilization with low-profile hardware and promotes accelerated rehabilitation while appearing to provide superior biomechanical strength to other methods.^13^ Future studies evaluating long-term outcomes and comparative fixation strength are warranted to define its role in the broader context of osteochondral lesion management.

## Disclosures

The authors declare the following financial interests/personal relationships which may be considered as potential competing interests: C.W.N. reports board or committee membership with the American Orthopaedic Society for Sports Medicine, American Academy of Orthopaedic Surgeons, and Arthroscopy Association of North America; receives other financial or material support from AO Foundation; is a paid presenter or speaker for Arthrex; reports editorial or governing board membership with *Arthroscopy*; receives publishing royalties and financial or material support from *Arthroscopy*; is a paid consultant for Guidepoint Consulting; and is a paid presenter or speaker for Vericel. S.F.D. reports a consulting or advisory relationship with Stryker Orthopaedics; receives speaking and lecture fees from AO North America; and receives funding grants from Orthopaedic Research and Education Foundation, Arthroscopy Association of North America, and Arthrex. All other authors (J.M.M., D.W.H.) declare that they have no known competing financial interests or personal relationships that could have appeared to influence the work reported in this paper.

## Supplementary Data

Video 1 Diagnostic arthroscopy in a right knee with a 30° arthroscope identifies a lateral femoral condyle osteochondral fracture. The lesion is subsequently removed and fixed via an open technique with knotless anchors and absorbable suture.
